# Effect of *Rumex Acetosa* Extract, a Herbal Drug, on the Absorption of Fexofenadine

**DOI:** 10.3390/pharmaceutics12060547

**Published:** 2020-06-12

**Authors:** Jung Hwan Ahn, Junhyeong Kim, Naveed Ur Rehman, Hye-Jin Kim, Mi-Jeong Ahn, Hye Jin Chung

**Affiliations:** College of Pharmacy and Research Institute of Pharmaceutical Sciences, Gyeongsang National University, Jinju 52828, Korea; anjh5803@naver.com (J.H.A.); jhk6914@naver.com (J.K.); naveed.rehman50@gmail.com (N.U.R.); black200203@gmail.com (H.-J.K.); amj5812@gnu.ac.kr (M.-J.A.)

**Keywords:** *P*-glycoprotein (*P*-gp), organic anion transporting polypeptide 1A2 (OATP1A2), *Rumex acetosa*, pharmacokinetics, fexofenadine, drug interaction

## Abstract

Herbal drugs are widely used for the auxiliary treatment of diseases. The pharmacokinetics of a drug may be altered when it is coadministered with herbal drugs that can affect drug absorption. The effects of herbal drugs on absorption must be evaluated. In this study, we investigated the effects of *Rumex acetosa (R. acetosa)* extract on fexofenadine absorption. Fexofenadine was selected as a model drug that is a substrate of *P*-glycoprotein (*P*-gp) and organic anion transporting polypeptide 1A2 (OATP1A2). Emodine—the major component of *R. acetosa* extract—showed *P*-gp inhibition in vitro and in vivo. Uptake of fexofenadine via OATP1A2 was inhibited by *R. acetosa* extract in OATP1A2 transfected cells. A pharmacokinetic study showed that the area under the plasma concentration–time curve (AUC) of fexofenadine was smaller in the *R. acetosa* extract coadministered group than in the control group. *R. acetosa* extract also decreased aqueous solubility of fexofenadine HCl. The results of this study suggest that *R. acetosa* extract could inhibit the absorption of certain drugs via intervention in the aqueous solubility and the drug transporters. Therefore, *R. acetosa* extract may cause drug interactions when coadministered with substrates of drug transporters and poorly water-soluble drugs, although further clinical studies are needed.

## 1. Introduction

Oral drug administration is a preferred route, offering the advantages of convenience and safety. Many drug interactions with foods and other drugs occur via alteration of drug absorption. There are absorptive transporters, such as organic anion transporting polypeptide (OATP) and secretory transporters, including *P*-glycoprotein (*P*-gp), associated with drug absorption. To improve drug therapy, it is necessary to investigate possible interactions mediated by transporters that could alter systemic exposure of drugs.

P-gp, belonging to the ATP binding cassette superfamily, is an ATP-dependent efflux protein that excretes drugs out of cells [1,2,3]. P-gp is an important factor limiting the absorption of drugs and plays a key role in drug distribution and resistance [3,4]. For example, P-gp overexpression induced by a hypoxic environment in many cancers decreases the effects of chemotherapy [5,6]. Furthermore, drug–drug interactions may occur when substrates of P-gp (e.g., cimetidine, digoxin, doxorubicin, fexofenadine, and vinblastine) are coadministered with inhibitors of P-gp (e.g., atorvastatin, ketoconazole and quinidine) or inducers of P-gp (e.g., rifampin and clotrimazole) [7,8]. The OATP family is also an important transporter for drug disposition. The OATP members of the solute carrier (SLC) family, contributes to the uptake of substrates, including endogenous compounds and drugs [9,10]. Drug–drug interactions and food–drug interactions mediated by these two active transporters—P-gp and OATP—have been reported. In addition, a study on medication use patterns revealed that 50% of 2590 study participants had taken at least one prescription drug during the week prior to the study, and 16% of them had taken one or more herbals/supplements [11,12]. Given that St. John’s wort was found to increase *P*-gp expression [13], it is necessary to evaluate the effects of herbal supplements on these transporters. Despite the widespread use of herbal drugs in combination with drugs, there has been little research on the interactions between drugs and herbal medicines.

This study investigated the effects of *Rumex acetosa (R. acetosa)* extract on *P*-gp and OATP1A2 in vitro and on fexofenadine absorption in vivo. *R. acetosa*, used in folk remedies for skin diseases, has been singled out as a natural herbal medicine for its potential to be used in combination with fexofenadine [14]. *R. acetosa* is widely distributed in eastern Asia and decoction of this plant has been used for the treatment of several health disorders such as fever, gastro-intestinal problems, inflammatory diseases. It is belonging in the Polygonaceae family, known to produce many biologic metabolites [15]. Particularly, *R. acetosa* is rich in anthraquinones and flavonoids that have anti-inflammatory and antiproliferative effects [16,17]. Emodin, a major anthraquinone component of *R. acetosa* extract, is reported that has the potential for *P*-gp mediated drug interaction [18] and has various pharmacological effects, such as antidiabetic [19] and anticancer activities [20].

Fexofenadine, a selective histamine H_1_ receptor antagonist, is widely used for seasonal allergic rhinitis and chronic idiopathic urticarial treatment [21]. There is no evidence for cardiotoxicity associated with fexofenadine, the active metabolite of terfenadine, even though terfenadine is not used anymore due to the risk of cardiac arrhythmia. Fexofenadine was selected as a model drug that is a marker substrate of *P*-gp [22] and OATP1A2 [23]. Fexofenadine is considered a good model drug, because only around 5% of its dose is metabolized and most of the dose is excreted into urine (11%) and feces (80%) as the unchanged form [24,25], which means that metabolism can be excluded in interpreting the pharmacokinetics of fexofenadine.

To date, there have been many drug interaction studies involving *P*-gp or OATP. However, there have been few studies concerning drug interactions with herbal medicines involving both *P*-gp and OATP1A2. Furthermore, it has been reported that the emodin acts on *P*-gp as an inducer [26] or an inhibitor [18]. Our results clarify the inhibitory effect of emodin on the *P*-gp through in vitro and in vivo study. In addition, our findings include the fact that *R. acetosa* extract could affect drug absorption via intervention in the OATP-mediated influx and the aqueous solubility. These results indicate that the effects of herbal medicines such as plant extracts, on drug absorption must be considered in terms of not only efflux through *P*-gp, but also OATP-mediated influx and the aqueous solubility.

## 2. Materials and Methods

### 2.1. Chemicals and Reagents

Fexofenadine hydrochloride and emodin were purchased from Tokyo Chemical Industry (Tokyo, Japan). Dimethyl sulfoxide (DMSO), terfenadine, verapamil, Dulbecco’s modified Eagle’s medium (DMEM) with high glucose, MEM non-essential amino acid solution (NEAA) and glutamine were purchased from Sigma-Aldrich (St. Louis, MO, USA). HPLC grade acetonitrile and water were purchased from Fisher Scientific Korea (Seoul, Korea). Emodin, emodin-8-*O*-β-d-glucoside, chrysophanol, chrysophanol-8-*O*-β-d-glucoside, physcion and physcion-8-*O*-β-d-glucoside isolated from *R. acetosa* were obtained from the pharmacognosy laboratory of the College of Pharmacy at Gyeongsang National University (Jinju, Korea) [27]. Fetal bovine serum (FBS), N-2-hydroxyethylpiperazine–N′-2-ethanesulfonic acid (HEPES) and Hanks’ balanced salt solution (HBSS) were purchased from Corning (Manassas, VA, USA). Penicillin–streptomycin, Opti-MEM and 0.25% (*w/v)* trypsin–EDTA were purchased from Gibco (Carlsbad, CA, USA). Phosphate buffered saline (PBS) was purchased from Welgene (Gyeongsan, Korea). An MDR assay kit (fluorometric) was purchased from Abcam (Cambridge, UK).

### 2.2. R. acetosa Extract

The *R. acetosa* extract was prepared by previously reported procedure [27]. Briefly, the dried whole part of *R. acetosa* was extracted with 70% ethanol. The extraction was performed by the Soxhlet extractor for 3 h at 80 °C. The extract was filtered and lyophilized.

The total phenol content and total flavonoid content of *R. acetosa* extract were 74.5 mg GAE (gallic acid equivalent)/g of dry weight and 180.3 μg QAE (quercetin equivalent)/g of dry weight, respectively. The contents of anthraquinones in *R. acetosa* extract were determined by HPLC. The contents of emodin, emodin-8-*O*-β-d-glucoside, chrysophanol, chrysophanol-8-*O*-β-d-glucoside, physcion and physcion-8-*O*-β-d-glucoside in *R. acetosa* extract were 0.94 ± 0.15%, 1.29 ± 0.06%, 0.68 ± 0.09%, 0.77 ± 0.12%, 0.17 ± 0.02% and 0.41% ± 0.05% (*w/w*), respectively. The values were expressed as mean ± standard deviation.

### 2.3. Cell Culture

The Caco-2 (HTB-37™) cells were purchased from the American Type Culture Collection (ATCC, Manassas, VA, USA). OATP1A2/SLCO1A2 transfected HEK293 cells were purchased from Corning (New York, NY, USA). The Caco-2 cells were cultured in high glucose added DMEM with 10% FBS, 1% NEAA, 10-mM HEPES, 4-mM glutamine, 100 U/mL of penicillin and 100 μg/mL of streptomycin, and maintained in humidified 5% CO_2_ at 37 °C. The medium of the Caco-2 cells was replaced 2–3 times per week.

The transfected HEK293 cells were cultured in high glucose added DMEM with 10% FBS and 1% NEAA, and maintained in 8% CO_2_ with low humidity at 37 °C for 4 h. After incubation for 4 h, the medium of the transfected HEK293 cells was replaced with high glucose added DMEM with 10% FBS, 1% NEAA and 2-mM sodium butyrate, and incubated for 24 h.

### 2.4. Cytotoxicity Assay

The cytotoxicity of *R. acetosa* extract on Caco-2 cells and HEK293 cells was measured using an EZ-Cytox cell viability assay kit (Daeil Lab Service, Seoul, Korea). The cells were cultured in DMEM containing 10% FBS, 1% NEAA, 10-mM HEPES, 100 U/mL of penicillin and 100-μg/mL streptomycin without phenol red. The seeding density was 3 × 10^4^ cells/well for Caco-2 cells and 2.5 × 10^4^ cell/well for HEK293 cells, respectively. The Caco-2 cells were incubated for 7 days and the HEK 293 cells were incubated for 24 h after seeding. The medium was replaced with 50 μL of new medium containing *R. acetosa* extract at the concentrations of 1, 2, 5, 10, 20, 50 and 100 μg/mL achieved the 0.5% of DMSO content. After 15 min of incubation, 5 μL of EZ-Cytox reagent (water-soluble tetrazolium) was added to the cells, and the cells were incubated for 3 h. Cell viability was calculated as a percentage of the absorbance at 450 nm compared to untreated cells.

### 2.5. P-gp Inhibition Test of Anthraquinones and R. acetosa Extract

The *P*-gp inhibition effect of anthraquinones from *R. acetosa* was evaluated via MDR assay kit using Caco-2 cells. It was reported that verapamil has concentration-dependent inhibition effects on absorptive and secretory transporters. Accordingly, 100-μM verapamil was used as a positive control [28]. Caco-2 cells were cultured in 96-well plates at a density of 5 × 10^5^ cells/mL and incubated in humidified 5% CO_2_ at 37 °C for 24 h. They were treated with 6 test compounds (10 μM) [18] or *R. acetosa* extract in HBSS and incubated for 15 min. The concentration levels of *R. acetosa* extract were 5, 10, 25 and 50 μg/mL. The MDR dye-loading solution was added at a volume of 100 μL and incubated. Fluorescence intensity was detected with a microplate reader Synerge H1 (Biotek, Winooski, VT, USA) at a wavelength of 490 nm for the excitation and 525 nm for the emission.

### 2.6. Fexofenadine Uptake Test Using OATP1A2/SLCO1A2 Transfected HEK293 Cells

The seeding density of the OATP1A2 overexpressed HEK293 cells was 10^5^ cells/well. Verapamil was used as a positive control with a concentration of 100 μM [28]. The cultured cells were washed twice with warmed HBSS with 5-mM MES after removing the medium, then 15-μM fexofenadine was treated with *R. acetosa* extract of 10, 20 and 50 μg/mL. After 15 min of incubation in 8% CO_2_ with low humidity at 37 °C, they were washed twice with cold HBSS. They were gently shaken after adding 120 μL of 50-ng/mL terfenadine in 80% acetonitrile. Terfenadine was used as an internal standard. After centrifugation at 10,000× *g* for 5 min, 50 μL of supernatant was mixed with 50 μL of 5-mM ammonium formate (pH 4). The liquid chromatography-tandem mass spectrometry (LC-MS/MS) was used to quantify the fexofenadine uptake amount [28,29].

### 2.7. LC-MS/MS Analysis

The chromatographic analysis was performed using an Agilent 1260 series (Agilent, Germany) HPLC system. Chromatographic separation was achieved from the Phoroshell^®^ column (C18, 3.0 × 50 mm, 2.7 µm). The mobile phase consisted of 5-mM ammonium formate (pH 4) in water (A) and acetonitrile (B). A gradient method was applied at a flow rate of 0.3 mL/min and, kept on the column temperature at 25 °C. The injection volume was 2 μL. An Agilent 6460 triple-quadruple mass spectrometer (Agilent Technologies, Singapore) with an electrospray ionization (ESI) source was used to detect the signal. It was operated in positive ion mode on multiple reaction monitoring (MRM). The monitored ions of fexofenadine and internal standard (terfenadine) were *m/z* 502→466 and *m/z* 472→436 [30,31], respectively. The collision energy and fragmentor of the ions were 25 V and 175 V for fexofenadine, and 25 V and 130 V for terfenadine, respectively. The data were acquired and processed using Mass Hunter Workstation B.06.00 software (Agilent Technologies, Singapore).

### 2.8. Animal Study

#### 2.8.1. Animals

Male Sprague‒Dawley rats (9 weeks, weighing 300 ± 50 g) were purchased from Koatech (Pyeongtaek, Korea). The rats were acclimated in the Animal Laboratory (Gyeongsang National University) under controlled condition of temperature (between 20 and 23 °C) and humidity (50% ± 5%) and allowed free access to food and water for 7 days. All rats were allowed to recover for 1 day after cannulation into the carotid artery. The rats were fasted for 12 h with free access to water, before the pharmacokinetic experiments.

#### 2.8.2. Pharmacokinetic Study

The pharmacokinetic study was performed on a rat model. The dose of *R. acetosa* extract evaluated was 2 g/kg, the maximum dose without the toxicity in rats (unpublished data). The selected oral dose of emodin was 11 mg/kg that inhibited *P*-gp mediated efflux in rats from the reported study [32]. All test compounds—including 11 mg/kg of emodin and 2 g/kg of *R. acetosa* extract suspended in 0.5% carboxy methyl cellulose (CMC)—were administered orally to rats. Same volume of 0.5% CMC was administered to the vehicle control group rats. After 30 min, a single dose of 10 mg/kg of fexofenadine in 10% ethanol was orally administered to each group of rats [33]. Blood samples of 120 μL were collected from the carotid artery at each time point (0, 0.25, 0.5, 0.75, 1, 1.5, 2, 4, 6, 8, 12 and 24 h) after oral administration of fexofenadine. The samples were then immediately centrifuged at 10,000× *g* and 4 °C for 10 min. All plasma samples were stored at −20 °C. The plasma concentrations of fexofenadine were determined by LC-MS/MS. All experimental procedures of the animal study were approved (GNU-170705-R0030, 5 July 2017) by the Animal Care and Use Committee of Gyeongsang National University, Korea.

#### 2.8.3. Sample Preparation

The method of sample preparation was a modified method of Isleyen et al. [34] for determination of fexofenadine plasma concentration. In summary, 50 μL of 50-ng/mL terfenadine in acetonitrile solution was added to a 50-μL aliquot of plasma, then 20 μL of aqueous 13-μM formic acid solution was added. After vortexing, 50 μL of extraction solvent (a mixture of dichloromethane, ethyl acetate, diethyl ether at the ratio of 30:40:30, *v/v/v*) was added. The sample was then vortexed for 40 s. The protein precipitation was performed via centrifugation at 10,000× *g* and 4 °C for 5 min. The supernatant was cooled at −80 °C for 10 min. The upper fraction of the supernatant was transferred to a polypropylene tube and evaporated with N_2_ gas. After being reconstituted with 200 μL of the mobile phase initial composition [5-mM ammonium formate (at a pH of 4): acetonitrile = 60:40], an aliquot of 2 μL was injected into LC-MS/MS.

### 2.9. Physicochemical Interaction Study

To investigate the possible physicochemical interactions between drug and *R. acetosa* extract, Fourier transform infrared (FT-IR) spectrum measurement and solubility test were carried out.

FT-IR spectra of fexofenadine HCl, *R. acetosa* extract and mixture of fexofenadine-extract (1:1) were measured by Nicolet iS 50 FT-IR spectrometer (Thermo Scientific, Waltham, MA, USA) with attenuated total reflectance (ATR) mode. 

The change on the solubility of fexofenadine after mixing with *R. acetosa* extract was tested. The method was modified previously reported method [35,36]. Briefly, 200 μg of fexofenadine and *R. acetosa* extract were placed in the tube after centrifugal vacuum evaporation of solvent. The control group has fexofenadine only, and the mixed group has both fexofenadine and the extract. A 200-μL aliquot of the simulated intestinal fluid (SIF, pH 6.8) without enzyme [37] was added to each tube. The tubes were then incubated in a shaking water bath at 37 °C for 12 h. The concentration of fexofenadine was 1 mg/mL, corresponding to the orally administered concentration to the rats (10 mg/5 mL/kg-fexofenadine with 5-mL/kg extract, total 10 mL). After the incubation, the tubes were centrifuged at 10,000× *g* for 10 min. The supernatant was filtered, diluted with mobile phase, and analyzed by LC-MS/MS.

### 2.10. Statistical Analysis

The statistical analysis was performed using one-way analysis of variance (ANOVA) followed by a Dunnett’s multiple comparison test. A *p*-value of less than 0.05 was considered statistically significant.

## 3. Results

### 3.1. Cytotoxicity Assay

**Figure 1:**
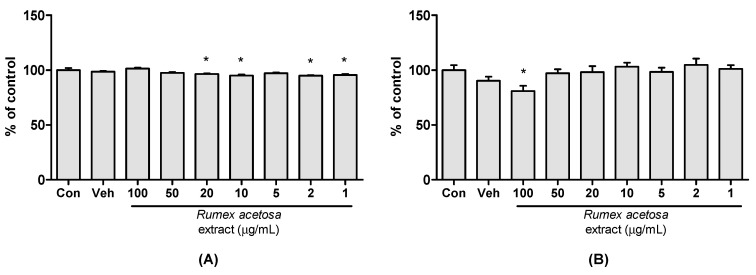
Cytotoxicity of *R. acetosa* extract in (**A**) Caco-2 cells and (**B**) HEK293 cells (*n* = 6). Con—media only treated control; Veh—vehicle treated group; *—*p* < 0.05 compared to media only treated control group.

Cell viability was expressed as a percentage of the absorbance value obtained from the media only treated control group (Figure 1). Even though there were statistically significant differences in Caco-2 cell viability between control and *R. acetosa* treated groups at concentrations of 20, 10, 2 and 1 μg/mL, the cell viability values were high enough to study (96.4% ± 1.3%, 95.0% ± 2.1%, 95.0% ± 1.2% and 95.5% ± 2.1% at concentrations of 20, 10, 2 and 1 μg/mL, respectively). It is suggested that there is a negligible cytotoxic effect of *R. acetosa* on the Caco-2 cells at the concentration range of 1 to 100 μg/mL.

There was no significant difference on the cell viability on HEK293 cells at the concentration ranges of 1 to 50 μg/mL. The cytotoxic effect of *R. acetosa* was only detected on HEK293 cells at a concentration of 100 μg/mL with the value of 80.9% ± 11.7%. This result suggests a dose window of *R. acetosa* extract for the experiment using HEK293 cells. It also indicates that the inhibitory effect of *R. acetosa* on fexofenadine uptake discussed in Section 3.2 was not due to the cytotoxic effects of *R. acetosa* on HEK293 cells at the concentration range tested.

### 3.2. P-gp Inhibition Test of Anthraquinones and R. acetosa Extract

**Figure 2:**
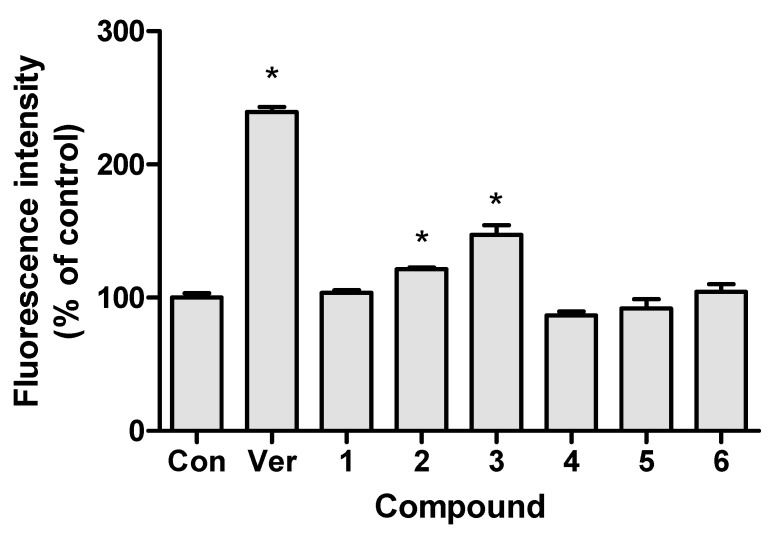
P-gp inhibitory effect of anthraquinones in Caco-2 cells. Cells were treated with 10-μM anthraquinones or 100-μM verapamil (*n* = 3). Con—vehicle treated control; Ver—verapamil; 1—chrysophanol; 2—chrysophanol-8-*O*-β-d-glucoside; 3—emodin; 4—emodin-8-*O*-β-d-glucoside; 5—physcion; 6—physcion-8-*O*-β-d-glucoside; *—*p* < 0.05 compared to control group.

To determine inhibitory effect of anthraquinones on *P*-gp, an MDR kit was used. The accumulated amount of fluorescent dye in the cells represented the *P*-gp inhibition activity. The measured fluorescence intensity is expressed as a percentage of the fluorescence intensity in the control group and is shown in Figure 2. The verapamil, chrysophanol-8-*O*-β-d-glucoside and emodin treated groups displayed significantly different fluorescence intensities in comparison to those of the control group. However, the chrysophanol-8-*O*-β-d-glucoside and emodin treated groups showed significantly higher fluorescence intensities than the control group, with average values of 121.4% ± 2.3% and 147.2% ± 12.4%, respectively (mean ± standard deviation). This result suggests that chrysophanol-8-*O*-β-d-glucoside and emodin affect the efflux of fluorescent dye from Caco-2 cells through *P*-gp inhibition. This is consistent with previous findings that emodin inhibits *P*-gp [18]. It is thus reasonable to suggest that herbal drug containing chrysophanol-8-*O*-β-d-glucoside and emodin may also inhibit *P*-gp.

**Figure 3:**
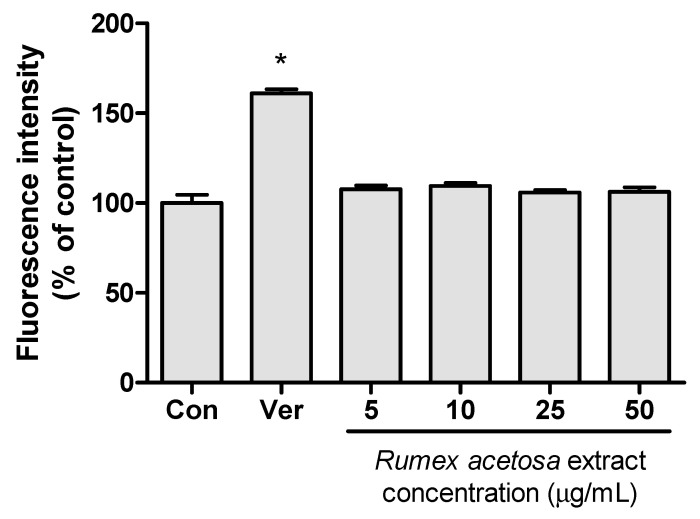
P-gp inhibition test of *R. acetosa* extract using an MDR kit in Caco-2 cells (*n* = 6). Con—vehicle treated control; Ver—100-μM verapamil; *—*p* < 0.05 compared to control group.

The effects of *R. acetosa* extract on the *P*-gp were also assessed using an MDR kit. The measured fluorescence intensity is expressed as a percentage of the fluorescence intensity in the control group and is shown in Figure 3. There was no significant difference in fluorescence intensity between the control and the *R. acetosa* extract treated group. The significant inhibitory effect at the 95% confidence interval was only detected in the verapamil group used as a positive control. Although *R. acetosa* extract contains chrysophanol-8-*O*-β-d-glucoside and emodin at concentrations of 0.77% ± 0.12% and 0.94% ± 0.15% (*w/w*), respectively [27], inhibitory effects on *P*-gp could not be detected from *R. acetosa* extract at the concentrations tested in this assay. The concentrations of emodin and chrysophanol-8-*O*-β-d-glucoside in *R. acetosa* extract may not be high enough to inhibit *P*-gp in Caco-2 cells.

### 3.3. Fexofenadine Uptake Test with OATP1A2/SLCO1A2 Transfected HEK293 Cells

**Figure 4:**
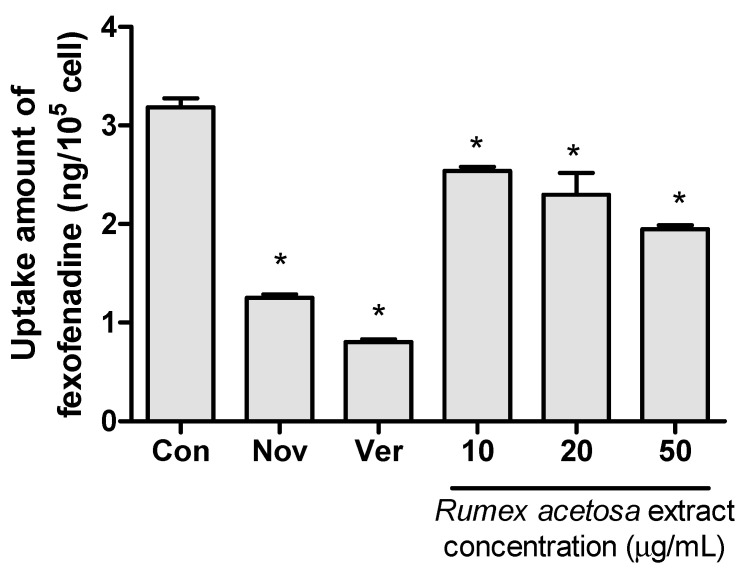
Inhibitory effect of *R. acetosa* extract on fexofenadine uptake in OATP1A2/SLCO1A2 transfected HEK293 cells (*n* = 6). Con—OATP1A2/SLCO1A2 transfected control; Nov—untransfected control; Ver—100-μM verapamil; *—*p* < 0.05 compared to control group.

The decreased fexofenadine uptake in the OATP1A2/SLCO1A2 transfected HEK293 cells represented the inhibitory effects on OATP1A2. The accumulated amount of fexofenadine in the OATP/SLCO1A2 transfected cells (the control group) was higher than that of untransfected cells (untransfected control group), which means that the OATP1A2 gene was transfected and expressed sufficiently in the control group. In addition, the fexofenadine uptake was lower in the verapamil cotreated group than in the transfected control group. In the *R. acetosa* extract co-treated group, the uptake amounts of fexofenadine in the OATP1A2 transfected HEK293 cells were significantly lower than that in the control group (Figure 4). This result suggests that *R. acetosa* extract could affect the absorption of fexofenadine through the inhibition of OATP1A2.

### 3.4. Pharmacokinetic Study

**Figure 5:**
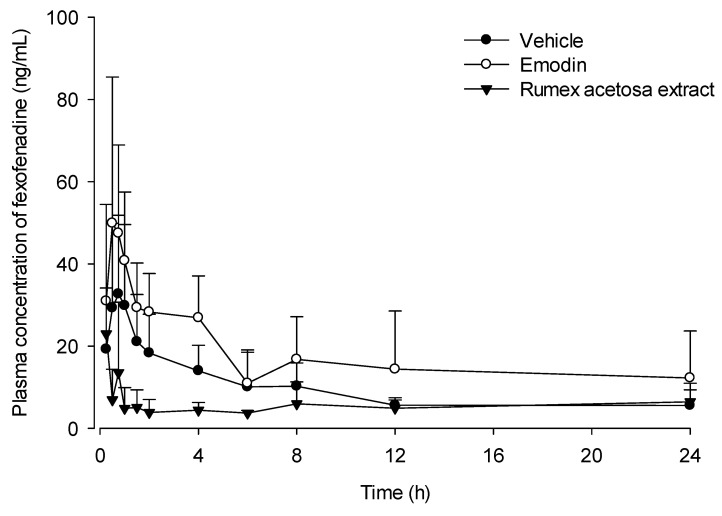
Mean plasma concentration–time profiles of fexofenadine (ng/mL) after oral coadministration of fexofenadine (10 mg/kg) with vehicle (●; *n* = 6), emodin (11 mg/ kg, ○; *n* = 6) and *R. acetosa* extract (2 g/kg, ▼; *n* = 6) to rats. Bars represent standard deviations.

The mean arterial plasma concentration–time profiles of fexofenadine after the pharmacokinetic study of fexofenadine in a rat drug interaction model, are shown in Figure 5. The pharmacokinetic parameters of fexofenadine after oral coadministration of vehicle, emodin and *R. acetosa* extract are shown in Table 1. The area under the plasma concentration–time curve (AUC) values of fexofenadine were 222.0 ± 85.5 ng∙h/mL in the control group and 411.9 ± 189.1 ng∙h/mL in the emodin coadministered group. In the emodin group, the absorption of fexofenadine was significantly higher, with a larger fexofenadine AUC and no difference in T_max_. However, the fexofenadine AUC was lower, with a value of 132.0 ± 50.5 ng∙h/mL, in the 2 g/kg *R. acetosa* extract coadministered group. Because fexofenadine is characterized by limited metabolism, it is probable that the lower fexofenadine AUC is due to the inhibitory effect of *R. acetosa* extract on absorption. Consequently, *R. acetosa* extract could inhibit the absorption of fexofenadine. Together with the results of the in vitro assay, this suggests that *R. acetosa* extract inhibits the intracellular uptake of fexofenadine via an intervention in OATP1A2.

### 3.5. Physicochemical Interaction Study

**Figure 6:**
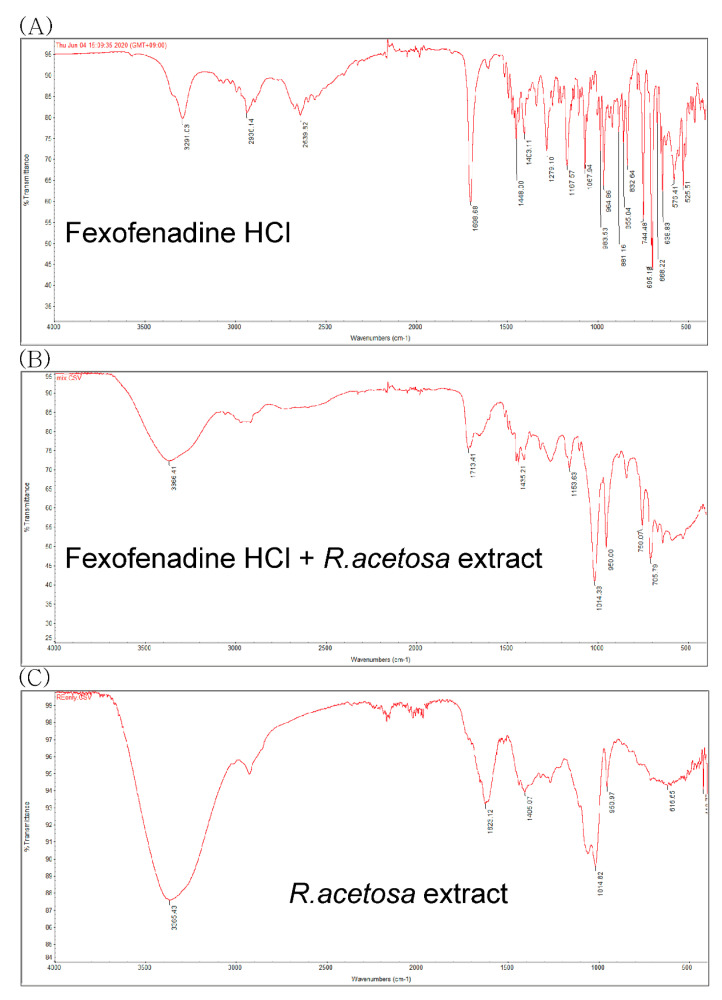
FT-IR spectra of (**A**) fexofenadine, (**B**) a mixture of fexofenadine and *R. acetosa* extract and (**C**) *R. acetosa* extract.

To evaluate the possible physicochemical interactions between *R. acetosa* extract and fexofenadine, FT-IR spectra of extract, fexofenadine and mixture were measured and are shown in Figure 6. The FT-IR spectrum of fexofenadine HCl showed the characteristic absorption bands at 3291.03 (OH stretching), 2936.14 (CH stretching), 2639.82 (OH of carboxylate), 1698.68 (CO stretching), 1448.00, 1403.11 (C=C stretching of aromatic ring), 1167.57 (CO stretching of tertiary alcohol) and 1067.94 (CO stretching of secondary alcohol) [38,39]. According to Figure 6, the mixture of *R. acetosa* extract and fexofenadine HCl showed the same bands compared to the pure fexofenadine HCl. It suggests that there is no significant physical interaction between fexofenadine molecule and *R. acetosa* extract component on fexofenadine functional groups.

However, there was significant difference on the solubility of fexofenadine after incubation with the extract (Table 2). The average solubilities of fexofenadine in SIF were 1.03 ± 0.04 mg/mL and 0.83 ± 0.10 mg/mL without and with *R. acetosa* extract, respectively. This result indicates that *R. acetosa* extract could alter the solubility of fexofenadine and lead to precipitation in gastro-intestinal tract.

## 4. Discussion

Pharmacokinetic drug interactions involving drug absorption should be considered for optimum drug therapy, apart from the drug interactions attributed to the oxidative metabolism via the CYP-450 system of different isozymes [40]. Ostensibly harmless natural products—such as juices, fruits, vegetables and herbal products in the form of ayurvedic medicine—have been reported in several studies to potentially cause many drug interactions affecting drug absorption mediated by transporters [41,42]. For example, emodin—a potential antineoplastic drug and a major component of the *Rhamnus, Rumex, Aloe*, *Rheum* and *Cassia* species—has been reported to be a possible *P*-gp inducer [26] or an inhibitor [18].

This study evaluated the effects of *R. acetosa* extract on the drug transporters discussed above, as well as its potential for drug interactions, while presenting a clear view of the interactions of emodin with the transporter *P*-gp. The major six anthraquinones present in *R. acetosa* were shown in our previous study [27]. A prior cytotoxicity assay was performed to establish the working range for the extract suitable for optimal viability of the cells during the experiment. Afterwards, the effects of these six anthraquinones on *P*-gp were demonstrated individually with an MDR assay kit using Caco-2 cells. Verapamil, being an inhibitor of *P*-gp, served as a positive control. Only groups treated with chrysophanol-8-*O*-β-d-glucoside and emodin showed higher fluorescence intensity than the control group, with average values of 121.4% ± 2.3% and 147.2% ± 12.4%, respectively, suggesting *P*-gp inhibition. This result is consistent with those obtained in a study by Min et al. [18], in which emodin was shown to inhibit *P*-gp. On the other hand, the results from the *P*-gp inhibition test of *R. acetosa* extract suggest no significant inhibition of the efflux transporter, as opposed to the emodin and chrysophanol-8-*O*-β-d-glucoside, which in contrast showed significant inhibition of the *P*-gp transporter when treated individually. A possible explanation is that the emodin content may not be high enough to exert its inhibitory effect in the extract. Chemical contents of herbal plant extracts can vary depending on various factors such as climate, harvesting seasons and extraction solvent. The probability of inhibition of *P*-gp by *R. acetosa* extract cannot be ruled out.

OATP1A2—the uptake transporter used in our in vitro test—is widely expressed in the intestines and serves as a major uptake mechanism for fexofenadine [43,44]. Sometimes, a substrate of *P*-gp—such as this study’s selected model drug, fexofenadine—can also be a substrate for the OATP uptake transporter [43,44], making it necessary to differentiate between the contributions of *P*-gp and OATP to potential drug interactions and those of other simultaneously administered drugs that could affect these transporters. Therefore, our in vitro studies were also performed with HEK293 cells transfected with the polypeptide transporter OATP1A2. *R. acetosa* extract was found to inhibit the uptake of fexofenadine through in vitro studies. In other words, the uptake of fexofenadine by OATP1A2 into cells declined when *R. acetosa* extract was used as a co-treatment. This result suggests that *R. acetosa* extract can affect the absorption of fexofenadine through the inhibition of OATP1A2.

A pharmacokinetic study was designed to verify the results of our in vitro study in view of the observed herbal extract’s drug interactions at the uptake transporter for fexofenadine in rats. Rat model is considered unsuitable to predict metabolic drug interaction in human [45]. However, there is a correlation in drug intestinal permeability with both carrier-mediated absorption and passive diffusion mechanisms between rat and human [46]. Because the property of our selected model drug, fexofenadine, has little metabolism, it is reasonable to use the rat model for predicting the intervention of extract on absorption. All rats were divided into 3 groups: an emodin administration group, an *R. acetosa* administration group and a control group. Eleven milligrams per kilogram of emodin, 2 g/kg of *R. acetosa* extract and 0.5% CMC as a control was administered orally to each group. Fexofenadine at the dose of 10 mg/kg was given orally to each group after 30 min. The results showed a smaller AUC of fexofenadine (132.1 ± 50.3 ng∙h/mL) in the *R. acetosa* group in comparison to that of the control group, in which the AUC was 222.0 ± 92.1 ng∙h/mL. These results suggest decreased absorption of fexofenadine in the rats treated with *R. acetosa* extract. In other words, the gut uptake transporter OATP1A2, which is responsible for fexofenadine absorption, was inhibited, as predicted by the in vitro results. Moreover, the alteration on the solubility of fexofenadine was also observed by *R. acetosa* extract through the physicochemical interaction study. The FT-IR spectra results suggest that there is no functional group interaction between fexofenadine and the component of *R. acetosa* extract. The fexofenadine solubility in SIF changed from 1.03 ± 0.04 mg/mL to 0.83 ± 0.10 mg/mL after mixing with the extract. It means that the solubility alteration could also be the reason for the decreased fexofenadine AUC by *R. acetosa* extract because fexofenadine HCl is Biopharmaceutics Classification System (BCS) class 3 drug with high solubility and low permeability. Drug interactions due to changes in solubility can be avoided by adjusting the administration time. *R. acetosa* extract contains many kinds of compounds, not only anthraquinones, but also flavonoids and polysaccharides [15]. They have also the possibility of interference with the drug absorption through the intervention to the transporters [47,48]. Particularly, one of the flavonoids of *R. acetosa* extract, epicatechin-3-*O*-gallate [49], also has an inhibitory effect on the OATP1A2 [50]. Moreover, there was the possibility that *R. acetosa* extract may change the gastric emptying time [51,52] and the pH in the gastro-intestinal tract when coadministered with the fexofenadine. The effects of anthraquinones on OATP have been rarely reported. Further studies are needed to elucidate the components in *R. acetosa* extract responsible for inhibition of fexofenadine absorption. Meanwhile, emodin increased the AUC for fexofenadine, possibly via the inhibitory effect on an efflux transporter of fexofenadine, *P*-gp [32], the effect of which on the uptake transporter of fexofenadine has yet to be fully understood.

Given the evidence from both in vitro and in vivo studies, *R. acetosa* extract should be used with caution when substrates of the drug transporters or poorly water-soluble drugs are prescribed.

## 5. Conclusions

The present study evaluated the effects of *R. acetosa* extract on 2 active transporters, P-gp and OATP1A2 and the resulting effects on fexofenadine absorption through in vitro and in vivo studies. The findings suggest that emodin can enhance fexofenadine absorption via an inhibitory effect on *P*-gp. In addition, *R. acetosa* extract could decrease the absorption of fexofenadine via intervention in the aqueous solubility and the drug transporters.

## Figures and Tables

**Table 1:** Pharmacokinetic parameters of fexofenadine after oral coadministration of fexofenadine (10 mg/kg) with vehicle (control), emodin and *R. acetosa* extract to rats. Values represent means ± standard deviations for AUC_0-24 h_ and C_max_ (ng/mL), median (range) for T_max_.

Parameters	Control (*n* = 6)	Emodin 11 mg/kg (*n* = 6)	*R. acetosa* Extract 2 g/kg (*n* = 6)
AUC_0-24 h_ (ng∙h/mL)	222.0 ± 85.5	411.9 ± 189.1 *	132.0 ± 50.5
C_max_ (ng/mL)	36.4 ± 22.8	53.4 ± 33.9	32.9 ± 28.5
T_max_ (h)	0.75 (0.5–1)	0.75 (0.5–1)	0.75 (0.25–8)

AUC_0-24 h_, total area under the plasma concentration–time curve from time zero to 24 h; C_max_—maximum plasma concentration; T_max_—time to reach C_max_; *—*p* < 0.05 compared to vehicle only control group.

**Table 2:** The solubility of fexofenadine HCl in simulated intestinal fluid (SIF) with and without *R. acetosa* extract.

Solubility	Without *R. acetosa* Extract(*n* = 3)	With *R. acetosa* Extract(*n* = 3)
Fexofenadine HCl concentration (mg/mL)	1.03 ± 0.04	0.83 ± 0.10 *

*—*p* < 0.05 compared to without *R. acetosa* extract.
